# The Effect of Mouthwash with *Sambucus williamsii* var. *coreana* Extract on Halitosis: A Randomised, Double-Blind, Placebo-Controlled Study

**DOI:** 10.3290/j.ohpd.b3240783

**Published:** 2022-07-22

**Authors:** Yu-Rin Kim, Seoul-Hee Nam

**Affiliations:** a Assistant Professor, Department of Dental Hygiene, Silla University, Busan, South Korea. Data curation, methodology, resources, validation, wrote the original draft, reviewed and edited the manuscript, read and agreed to the published version of the manuscript.; b Associate Professor, Department of Dental Hygiene, College of Health Sciences, Kangwon National University, Samcheok, South Korea. Conceptualisation, data curation, methodology, supervision, wrote the original draft, reviewed and edited the manuscript, read and agreed to the published version of the manuscript.

**Keywords:** halitosis, mouthwash, oral health promotion, *Sambucus williamsii* var. *coreana* extract

## Abstract

**Purpose:**

To clinically assess the effects of a mouthwash containing an extract of *Sambucus williamsii* var. *coreana*, a natural ingredient, on halitosis and halitosis-causing bacteria.

**Materials and Methods:**

This randomised, double-blind, placebo-controlled study included 77 participants. The experiment was conducted at Misoplant Dental Clinic in Busan, South Korea. Participants were divided into two groups: a control group consisting of 38 participants, to who whom a saline gargle was administered 1x/day for 30 s, and an experimental group consisting of 39 participants, who used a mouthwash containing *Sambucus williamsii* var. *coreana* extract 1x/day for 30 s for 1 day or 5 days. Halitosis was measured for 30 s in the absence of brushing or moisture. A sterile #15 paper point was placed in the gingival sulcus for 10 s, after which it was analysed using the polymerase chain reaction (PCR) method. The measurement time points were defined as follows: ‘baseline’: before starting the gargling routine; ‘1 day of treatment’: immediately after gargling once on day 1; ‘5 days of treatment ’: after gargling once a day for 5 days. Clinical results were statistically analysed using SPSS 24.0 for Windows.

**Results:**

Compared to the control group, halitosis clearly decreased in the experimental groups ‘5 days of treatment’ and ‘1 day of treatment’ (p < 0.05). There were statistically significant differences in the levels of halitosis-causing bacteria between the two groups; bacterial concentration in the experimental group decreased statistically significantly from day 1 to day 5 day of gargling with *Sambucus williamsii* var. *coreana* extract (p < 0.05). Accordingly, we clinically verified that *Sambucus williamsii* var. *coreana* has antibacterial effects against oral bacteria.

**Conclusion:**

The application of *Sambucus williamsii* var. *coreana* extracts effectively reduced halitosis and halitosis-causing bacteria. Therefore, mouthwashes containing *Sambucus williamsii* var. *coreana* extract can be used as an effective substitute for chemical formulations for treating halitosis.

The COVID-19 pandemic has resulted in the modification of various life style behaviours to promote better infection control. To prevent infection, toothbrushing is restricted in public facilities such as schools and workplaces; thus, the demand for mouthwashes as an oral health care aid is rapidly increasing.^28^ Various chemical ingredients are found in commercially available mouthwashes. Chlorhexidine, one of the most commonly used chemical mouthwashes, has been reported to cause discolouration of teeth and soft tissues, as well as dysgeusia when used for prolonged periods.^14^ In addition, the alcohol included in some mouthwashes to provide a refreshing feeling causes xerostomia along with a possible increase in oral microorganisms and halitosis.^12,35^ Moreover, long-term use of mouthwashes containing chemical components can harm the human body and favour development of resistant bacteria. Because of these problems, natural medicines are becoming safe and effective alternatives to prevent and treat oral diseases.^1^ Various studies have been done worldwide on natural products with pharmacological activity.^18^ In South Africa and India, essential oils extracted from plants have antibacterial, anti-inflammatory and antifungal effects, so they are used in traditional medicine.^9,34^ Forsythiae fructus extract,^7^ mulberry leaf extract,^6^ bamboo leaf extract,^15^ and cinnamon extract^1^ are natural antibacterial agents effective in the prevention of oral diseases. *Sambucus williamsii* var. *coreana* is a deciduous broad-leaved shrub distributed throughout South Korea, China, and Japan. It grows in shady, humid mountain valleys. In traditional oriental medicine, *Sambucus williamsii* var. coreana is used as a medicine for bone health, musculoskeletal strain, kidney disease, and as diuretic for edema.^25^ Most of the existing studies on Sambucus williamsii var. coreana are studies on physiological activity.^5,23,36^ There are few studies dealing with *Sambucus williamsii* var. *coreana* concerning oral diseases, and none have clinically verified *Sambucus williamsii* var. *coreana* effects on suppression and improvement of halitosis.

Studies have reported a possible association between the presence of periodontitis and the risk of COVID-19 infection and subsequent complications.^30^ During the pandemic, there has also been an increase in the incidence of halitosis, especially due to prolonged usage of face masks.

Decomposition of food debris and proteins by oral bacteria results in halitosis.^19^ Volatile sulfur compounds (VSCs), a component of halitosis, are associated with gram-negative anaerobic oral bacteria.^17,27^ In particular, anaerobic bacteria such as *Fusobacteria nucleatum (F. nucleatum), Treponema denticola (T. denticola), Prevotella intermedia (P. intermedia)* and *Porphyromonas gingivalis (P. gingivalis)* are known to produce methionine, cysteine, methylmercaptan and hydrogen sulfide from serum proteins.^26,32^ *P. intermedia* and *F. nucleatum* are found only in patients with halitosis who also had moderate to severe periodontitis.^10^ Management of plaque in the oral cavity, by inhibition or elimination of bacteria, is important for maintaining the health of the gingival tissue.^8^

This points to the need to verify the antibacterial effect of mouthwashes to prevent or reduce halitosis. This study aims to assess the possible inhibitory effects of *Sambucus williamsii* var. *coreana* extract on halitosis-causing bacteria and its influence on the overall oral health of patients. In addition, we aimed to assess the effects of the mouthwash containing *Sambucus williamsii* var. *coreana* extract on halitosis.

## Materials and Methods

### Study Participants

**Fig 1:**
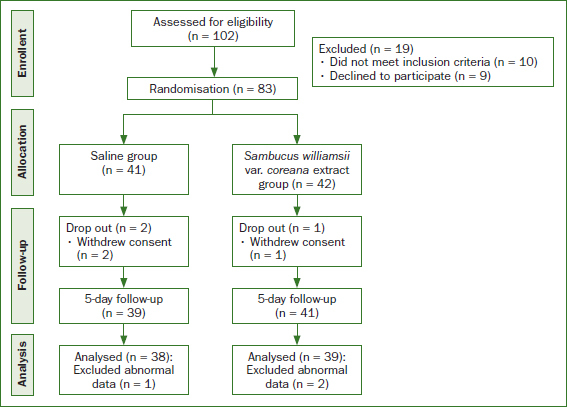
Flowchart of study method

This study followed the guidelines of the International Council for Harmonisation of Technical Requirements for Pharmaceuticals for human use (ICH). The Silla University Institutional Review Board (1041449-202008-HR-001, Busan, South Korea) approved this study, and it was registered on the WHO International Clinical Trial Registry Platform (registration number: KCT0007064; https://cris.nih.go.kr/cris/search/detailSearch.do/21436). All participants were informed of the objective and procedures of the study. Participants were also informed that refusing to participate would not be penalised, and withdrawal from the study was allowed at any time. Written consent was obtained from all participants before enrolment. The sample size was calculated using the G* Power 3.1 program. Sixty-eight participants were required for an independent t-test with a statistical significance level of α = 0.05 in a two-sided test, power = 0.8, and effect size = 0.7. The initial sample size was planned to be 96 with an anticipated dropout rate of 40%; the actual number of participants in this study was 100. A high dropout rate was set considering that the participants were college students or working adults. Among the 102 subjects who were assessed, 19 participants did not meet the inclusion criteria or refused to participate. The remaining 83 participants were randomly assigned to the control group or the experimental group. Additionally, six participants were excluded from the 5-day intervention phase. Finally, data from a sample of 77 participants were analysed in this study (Fig 1).

### Extraction of *Sambucus williamsii* var. *coreana*

We purchased *Sambucus williamsii* var. *coreana* (grown in Goesan, Chungcheongbuk-do, South Korea) from Cheongmyeong (Chungju-si, Chungcheongbuk-do, South Korea) and made the extract as follows: 80% ethanol was added to 100 g of dried *Sambucus williamsii* var. *coreana* stems, and this was processed in a heating mantle at 60°Ϲ for 12 h. The concentrated *Sambucus williamsii* var. *coreana* extract was prepared using a rotary vacuum evaporator (N-1300E.V.S. EYELA, Tokyo Rikakikai; Tokyo, Japan) after filtering it through filter paper (Advantec No. 2; Tokyo, Japan). This concentrated *Sambucus williamsii* var. *coreana* extract was then lyophilised in a freeze dryer (Ilshin Lab; Dongducheon-si, Gyeonggi-do, South Korea) to obtain the *Sambucus williamsii* var. *coreana* powder. This powder was further diluted in sterile distilled water and used in a mouthwash at a concentration of 10 mg/ml.

### Study Design and Protocol

This study was a randomised, double-blind, placebo-controlled trial. The participants who agreed to participate in the study visited the Misoplant Dental Clinic in Busan between October 2020 and June 2021. A dental hygienist with more than 10 years of experience explained the importance and purpose of the study. Among those with 16 or more healthy remaining teeth, participants with the following diseases were excluded from the study: severe periodontitis, one or more carious lesions, dry mouth, systemic diseases that can cause halitosis (eg, liver disease, kidney disease, Sjögren’s disease syndrome, rheumatism), smoking, sinusitis, rhinitis. A final sample of 77 participants was analysed.

### Clinical Examination

The study participants visited Misoplant Dental Clinic in Busan and were orally examined by a dentist. Two trained dental hygienists performed light scaling to ensure the homogeneity of oral conditions among the participants, after which the participants had a recovery time of 1 week. To establish as closely as possible the same oral environment, each participant received the same type of toothbrush and toothpaste, and they learned the appropriate brushing technique prior to participating in the study. In addition, dietary counseling was provided and participants were instructed to avoid eating foods that can induce halitosis, such as garlic, onions, coffee, and curry. One week after scaling was set as the ‘baseline’ time point. Without having performed any oral hygiene practices, participants visited the dentist in the morning on an empty stomach to measure bad breath. Oral bacteria causing bad breath were collected from buccal surfaces of the maxillary right first molar (#16) and mandibular left first molar (#36). The mouthwashes provided to the participants were labeled such that it was not known whether the participants were in the experimental group or the control group. In order to blind the participants to the placebo group, the solution was provided in a non-transparent brown container. For five days, the experimental group was instructed to gargle with 15 ml of *Sambucus williamsii* var. *coreana* extract for 30 s before going to sleep, and the control group was instructed to gargle with 15 ml of saline for 30 s. The total study duration was 5 days. All participants underwent clinical examinations – measuring bad breath and microbiological analysis – after waking up and without any oral hygiene practice or moisture in the mouth.

### Clinical Measurement Tool

#### Questionnaire

Age, marital status and information on systemic diseases were included in a questionnaire considering general characteristics. The participants answered the questions about frequency and time of toothbrushing. In order to gather data on oral health status, the modified and supplemented questionnaire of a previous study was used.^11,13,16,24^ The questionnaire consisted of 10 items, and scoring was based on a 5-point Likert scale, in which a higher score indicated better oral health status. The reliability of the questionnaire was 0.319. Pseudo-halitosis (subjective bad breath) was also assessed on a 5-point scale; the higher the score, the stronger the bad breath. A 5-point scale was also used to measure the degree of improvement in halitosis after using the mouthwash; a higher score indicated lesser halitosis. The reliability of the questionnaire was 0.853.

#### Halitosis measurement

Halitosis was measured with the Refres device (Mattz; Tokyo, Japan). The participants kept their mouths closed for a minimum of 5 min without brushing their teeth, and kept their mouths dry to collect VSCs in the oral cavity. The results were recorded accordingly. After breathing only through the nose for 3 min, a mouthpiece fitted on a probe of the halitosis detector was inserted into the participant’s mouth for 30 s to measure halitosis. The criteria for determining bad breath were: ‘no bad breath’ up to 30 BBV (bad breath value); ‘slight bad breath’ up to 50 BBV; ‘strong bad breath’ up to 70 BBV; ‘very strong bad breath’ up to 90 BBV; ‘severe bad breath’ up to 100 BBV (Table 1) The normal range is a BBV of 30-50, and treatment is indicated in those with a BBV >50.

**Table 1:** Classification of BBV

BBV	Halitosis degree
~30 BBV	Normal
~50 BBV	Slight
~70 BBV	Strong (needing treatment)
~90 BBV	Very strong (needing treatment)
~100 BBV	Severe (needing treatment)
101 BBV ~	Unmeasurable (needing treatment)

#### Microbiological analysis

After inserting sterilised #15 paper points into the gingival sulcus of two posterior teeth (#16 and #36) for 10 s in each participant with pocket depths <4, the paper points were placed in 1.5-ml tubes. The tubes were instantly stored at -20°C until just before analysis. DNA was extracted from the collected paper points by an AccuPrep Universal RNA Extraction Kit (Bioneer; Daejeon, South Korea) following manufacturer’s instructions. OligoMix (YD Global Life Science; Seongnam, South Korea) and three oligonucleotides (forward primer, reverse primer, and probe) (Table 2) that react specifically to each bacterium were used.^20^ We combined the following to prepare a reaction sample for the polymerase chain reaction (PCR): 9 µl of OligoMix, 10 µl of 2x probe qPCR mix (Takara Bio; Shiga, Japan), and 1 µl of template DNA. A 96-well plate with the PCR reaction sample was placed in the CFX96 Touch Real-Time PCR Detection System (Bio-Rad; Hercules, CA, USA) for DNA amplification. The conditions of PCR were as follows: initial denaturation at 95°C for 30 s, denaturation at 95°C for 10 s, and annealing for 30 s at 62°C with 40 repeated cycles. The Bio-Rad CFX Manager Software program calculated the cycle threshold (Ct) value. The number of copies was derived by plotting the Ct value in the standard curve of each bacterium.

**Table 2:** Primers and probes used in the real-time PCR assays (6 types)

Bacteria	Target genes	Primers/Probe sets	Amplicon size (bp)
*Porphyromonas gingivalis*	Hemagglutinin (phg) gene	5′-ACACGGTGTATCGTGACGGC-3’ 5′-GCCGGCTGCGTACTTAACCT-3’ 5′-HEX-CGACCTACCGCGATGCAGGA-BHQ1–3’	119
*Treponema denticola*	Oligopeptidase B (opdB) gene	5′-AGAAAGGCTTTGGGCGACAG-3’ 5′-GCTGGAGCCGTAGCTTCCAT-3’ 5′-Cy5-CGGGTCCTCACCCGCTCTTC-BHQ2–3’	127
*Fusobacterium nucleatum*	16S ribosomal RNA gene	5′-GGCTGTCGTCAGCTCGTGTC-3’ 5′-CTCATCGCAGGCAGTATCGC-3’ 5′-FAM-AACGAGCGCAACCCCTTTCG-BHQ1–3’	114
*Prevotella intermedia*	Hemagglutinin (phg) gene	5′-CACACGCTGGCGAAACCTAC-3’ 5′-CACGTGGCGTTGCTTCTTTC-3’ 5′-HEX-CCGAAGATGCGCCGTTGAAC-BHQ1–3’	143
*Prevotella nigrescens*	Gyrase subunit B (gyrB) gene	5′-AGCAAGCTGTAGGCGAGGCT-3’ 5′-GCTGAACACTTTCGCGTGCT-3’ 5′-Texas Red-GCTCGTATTGCAGCCCGCAA-BHQ2–3’	132
*Campylobacter rectus*	groEL gene	5′-AAATTTAAGCGGCGACGAGG-3’ 5′-TCCTTGCTCACGCTTACGGA-3’ 5′-HEX-GGCTTTGACGCGGGCGTAGT-BHQ1–3’	132

### Statistical Analysis

All data were analysed using SPSS 24.0 for Windows (IBM; Armonk, NY, USA). Frequency analysis was employed when analysing the demographic characteristics of the two groups. The independent t-test and Fisher’s exact test were performed to compare the differences in oral-health behaviours and oral health status between the groups. The independent t-test and one-way ANOVA were carried out on the measurement results of halitosis and microbiological analysis (the clinical indicators) at a significance level of p = 0.05 to evaluate changes over time according to duration of gargling (1 vs 5 days). The post-hoc Tukey’s test was performed to analyse differences between the groups.

## Results

### Participant Characteristics

There were more women than men in both the control group and the experimental group. The average age of participants in the control group was 37.5 years, and that of the participants in the experimental group was 38.6 years. The number of married and single participants was similar, and there were more people without systemic disease in both groups. None of the variables differend statistically significantly between the two groups (Table 3).

**Table 3:** Participant characteristics of the control and experimental groups

Characteristics		N (%)		*p-value
		Saline group (n=38)	*Sambucus williamsii* var. *coreana* extract group (n=39)	
Gender	Male	15 (39.5)	13 (33.3)	0.575
	Female	23 (60.5)	26 (66.7)	
^¥^Age (mean ± SD)		37.53 ± 16.93	38.56 ± 17.90	0.795
Marital status	Single	19 (50.0)	19 (48.7)	0.910
	Married	19 (50.0)	20 (51.3)	
Systemic disease	No disease	34 (89.5)	35 (89.7)	0.969
	Have a disease	4 (10.5)	4 (10.3)	

^¥^p-values were determined by the independent t-test, *p-values were determined by the Chi^2^ test (p < 0.05); values are means ± standard deviations.

### Differences in Oral Health Practices, Oral Health Status and Halitosis Status between the Two Groups

There was no statistically significant difference between the two groups in overall oral health behaviour, oral health status items, presence or absence of bad breath, and severity of bad breath (p > 0.05). However, the degree of improvement in halitosis after gargling was statistically significantly higher in the experimental groups than in the control group (p < 0.05) (Table 4).

**Table 4:** Oral health practices and oral health status according by group

	Characteristics	Control (saline = group) (n = 38)	*Sambucus williamsii* var. *coreana* extract group (n=39)	*p-value
Oral health behaviour	^¥^Frequency of brushing per day	3.68 ± 0.67	3.72 ± 0.69	0.827
	I brush my teeth before breakfast. (n = 54)	26 (48.1)	28 (51.9)	0.746
	I brush my teeth after breakfast. (n = 51)	23 (45.1)	28 (54.9)	0.296
	I brush my teeth after lunch. (n = 52)	26 (50.0)	26 (50.0)	0.869
	I brush my teeth after dinner. (n = 48)	23 (47.9)	25 (52.1)	0.746
	I brush my teeth before going to bed. (n = 38)	19 (50.0)	19 (50.0)	0.910
Oral health status	^¥^I have good oral health.	2.87 ± 0.62	2.79 ± 0.61	0.603
	^¥^I’ve never had a hard time chewing food.	3.03 ± 1.35	3.15 ± 1.29	0.672
	^¥^I have never had swollen or bleeding gums.	3.13 ± 1.04	3.08 ± 0.96	0.811
	^¥^I don’t bleed even when I brush my teeth.	4.03 ± 0.85	3.95 ± 0.83	0.686
	^¥^I like to eat cold or hot food.	3.66 ± 1.07	3.51 ± 0.99	0.540
	^¥^My mouth doesn’t feel dry.	2.87 ± 0.96	2.90 ± 0.94	0.894
	^¥^I have no halitosis.	2.76 ± 0.88	2.79 ± 0.86	0.874
	^¥^I have correct pronunciation.	3.24 ± 0.85	3.38 ± 0.82	0.439
	^¥^I don’t have any discomfort in the jaw joint.	3.76 ± 1.15	3.87 ± 1.29	0.677
	^¥^I am not concerned about my oral health.	3.05 ± 1.01	3.10 ± 1.05	0.832
Halitosis	I have halitosis (n = 69)	34 (49.3)	35 (50.7)	0.969
	^¥^Degree of severity in halitosis	2.29 ± 1.11	2.28 ± 1.12	0.977
	My halitosis has decreased (n = 53)	17 (32.1)	36 (67.9)	0.000
	¥Degree of reduction in halitosis	2.89 ± 0.95	4.08 ± 0.48	0.000

*p-values were determined by independent t- test, ^¥^Fisher’s exact test (p < 0.05); values are means ± standard deviations.

### Changes in Bad Breath after Gargling

The experimental and control groups showed considerable differences. There was no statistically significant difference in the control (saline) group over time (p > 0.05). However, the two experimental subgroups (day 1 vs day 5) differed statistically significantly. These findings confirm that the application of the mouthwash containing the *Sambucus williamsii* var. *coreana* extract reduced the occurrence of halitosis, with a greater reduction after 5 days than after 1 day of gargling (p < 0.05) (Table 5).

**Table 5:** Changes in halitosis based on gargling routine

Variables	Mean ± SD			*p-value
	Baseline	1 day of treatment	5 days of treatment	
Saline group	37.17 ± 7.71^a^	33.20 ± 9.02^a^	30.20 ± 7.74^a^	0.298
*Sambucus williamsii* var.* coreana* extract group	37.00 ± 10.11^a^	23.33 ± 9.31^b^	11.86 ± 5.43^c^	0.000
^¥^p-value	0.971	0.048	0.000	

^¥^p-values were determined by the independent t-test, *p-values were determined by one-way ANOVA and the post-hoc Tukey test (p < 0.05); values are mean ± standard deviations; the unit of measure is BBV. Different superscript letters indicate statistically significant difference (post-hoc Tukey test).

### Antibacterial Effect against Bacteria Causing Bad Breath

*P. gingivalis* and *T. denticola* showed statistically significant differences in the experimental groups (p < 0.05) after 1 day vs 5 days of gargling with the *Sambucus williamsii* var. *coreana* extract. The levels of *F. nucleatum* and *Campylobacter rectus* (*C. rectus*) differed statistically significantly only in the ‘1 day of treatment’ subgroup (p < 0.05), while the levels of *P. nigrescens* showed a statistically significant difference only in the ‘5 days of treatment’ subgroup (p < 0.05); there was no statistically significant difference in the levels of *P. intermedia* between the two groups (p > 0.05). In the control group, only *F. nucleatum* statistically significantly decreased between day 1 and day 5 of gargling with saline. In the experimental group, the levels of *P. gingivalis, T. denticola, F. nocleatum, C. rectus,* and *P. intermedia* statistically significantly decreased as the duration of the gargling routine increased (Table 6).

**Table 6:** Changes in halitosis-causing oral bacteria with gargling routine

Variables	Group	Mean ± SD			
		Baseline	1 day of treatment	5 days of treatment	*p-value
*Porphyromonas gingivalis*	Saline	139.68 ± 136.89^a^	127.07 ± 111.35^a^	143.15 ± 118.46^a^	0.931
	*Sambucus williamsii* var.* coreana* extract	116.02 ± 99.59^a^	9.17 ± 8.49^b^	8.33 ± 7.02^b^	0.000
	^¥^p-value	0.597	0.000	0.000	
*Treponema denticola*	Saline	5.25 ± 9.01^a^	3.20 ± 5.31^b^	4.03 ± 6.66^b^	0.733
	*Sambucus williamsii* var.* coreana* extract	5.72 ± 7.99	0.00 ± 0.000^b^	0.00 ± 0.00^b^	0.002
	^¥^p-value	0.881	0.023	0.022	
*Fusobacterium nucleatum*	Saline	343461.52 ± 22431.09^a^	292977.51 ± 228005.08a	99815.55 ± 97770.28^b^	0.004
	*Sambucus williamsii* var.* coreana* extract	299644.85 ± 267608.34^a^	116458.66 ± 143745.98^b^	52501.04 ± 67242.74^b^	0.002
	^¥^p-value	0.635	0.017	0.161	
*Prevotella intermedia*	Saline	2364.93 ± 3499.51^a^	2208.70 ± 4699.11a	375.25 ± 482.66a	0.247
	*Sambucus williamsii* var.* coreana* extract	20877.65 ± 1059.54^a^	900.93 ± 2248.17^b^	111.90 ± 151.02^b^	0.049
	^¥^p-value	0.117	0.335	0.051	
*Prevotella nigrescens*	Saline	995.48 ± 840.02^a^	802.32 ± 685.51^a^	653.65 ± 265.92^a^	0.411
	*Sambucus williamsii* var.* coreana* extract	1026.47 ± 1059.54^a^	664.47 ± 1137.39^a,b^	207.88 ± 220.59^b^	0.080
	^¥^p-value	0.933	0.704	0.000	
*Campylobacter rectus*	Saline	120.53 ± 124.88^a^	86.55 ± 107.29^ a^	69.98 ± 100.21^a^	0.452
	*Sambucus williamsii* var.* coreana* extract	127.47 ± 143.85^a^	5.05 ± 6.96^b^	21.53 ± 53.88^b^	0.000
	^¥^p-value	0.889	0.005	0.107	

^¥^p-values were determined by independent t-test; *p-values were determined by one-way ANOVA and post-hoc Tukey’s test (p < 0.05); values are means ± standard deviations; the unit of measure is Ct. Different superscript letters indicate statistically significant difference (post-hoc Tukey test).

## Discussion

Oral health is an essential component of overall health, as it is related to digestion and nutritional intake. Oral hygiene is also reported to be an important factor in health indicators.^22^ Halitosis is the malodor of exhaled breath generated by the oral environment or systemic disease, causing discomfort to oneself or others.^4^ Halitosis is not only an indicator of oral health and general health, but it also affects interpersonal relationships and adversely affects social life and mental health; therefore, efforts to reduce halitosis are needed.^33^

In approximately 90% of halitosis cases, the cause is found in the oral cavity. VSCs, the cause of the odor, are generated when bacteria decompose the proteins contained in dental plaque, tongue plaque, saliva, and food residues.^3,4^ Therefore, it is crucial to reduce the bacteria that cause halitosis, and a general method for this is brushing. But when brushing is not possible, mouthwash is widely used as a convenient alternative.^21^ In addition, the use of mouthwashes is also gaining popularity due to the spread of COVID-19. Therefore, we evaluated whether the natural ingredient *Sambucus williamsii* var. *coreana* extract could play a role as an active agent in a mouthwash to reduce halitosis and halitosis-causing bacteria, without the possible side effects of chemical components.

The present findings indicate an improvement in subjective halitosis among the experimental group. In the control group, the average improvement was 2.89, whereas the average improvement in the experimental group was 4.08. In addition to the reduction in subjective halitosis, the clinically measured halitosis score decreased over time in ‘treatment’ and ‘after 5 days’ in the experimental group, owing to the inhibitory effect of the extract on halitosis-causing bacteria. Although many studies reported the antibacterial effect of *Sambucus williamsii* var. *coreana*,^13-16^ no studies verified its efficacy against bad breath, so the clinical results of this study are meaningful in terms of practicality for improving bad breath.

The oral microflora consists of approximately 700 types of bacteria. Among them, *P. gingivalis, P. nigrescens, P. intermedia*, and *T. denticola* are highly related to halitosis, and the patients with severe halitosis have more *T. forsythia*.^31^ Accordingly, it has been reported that cinnamon extract was effective against *P. gingivalis*,^29^ and Jung et al^15^ verified that bamboo leaf extract was effective against *T. denticola. T. denticola* is a bacterium that attaches to gingival epithelial cells and induces inflammatory cytokine production in gingival fibroblasts to advance early periodontitis.^2^ *T. denticola* is associated with ulcerative gingivitis and acute periodontitis, and causes secondary infections such as diabetes, hypertension, coronary artery stenosis, coronary artery thrombosis, and syphilis (bacterial vaginitis).^29^ In this study, *T. denticola* was not detected after application of *Sambucus williamsii* var. *coreana* extract. Therefore, *Sambucus williamsii* var. *coreana* extract can be very useful in reducing the levels of *T. denticola*, which is involved in periodontal disease as well as halitosis. In addition, the levels of *P. gingivalis, F. nucleatum, P. intermedia,* and *C. rectus* were statistically significantly lower in the experimental group than in the control group. Thus, mouthwash containing *Sambucus williamsii* var. *coreana* extract suppresses and reduces the occurrence of halitosis by inhibiting bacteria that cause bad breath.

Halitosis has become a common problem in recent months owing to the prolonged use of masks due to COVID-19, having an effect on interpersonal relationships and causing discomfort in social situations. Therefore, halitosis is an important factor to be considered in oral health management, and mouthwashes should be effective in fighting bad breath. This study proves that the mouthwash containing *Sambucus williamsii* var. *coreana* extract had an excellent effect on reducing bad breath. Therefore, *Sambucus williamsii* var. *coreana* extract can be used as a natural substance in a mouthwash. A component analysis showing the effect of the *Sambucus williamsii* var. *coreana* extract will be performed in future research. Additional research with a sample that includes children and pregnant women is needed to assess the safety of *Sambucus williamsii* var. *coreana* extract as an antibacterial agent in a mouthwash and make it widely available to the population at risk.

## Conclusion

The increased incidence of halitosis, due to prolonged use of mouth masks during the COVID-19 pandemic, has resulted in an increased demand for an effective mouthwash. The findings of this study confirm that a mouthwash containing *Sambucus williamsii* var. *coreana* extract effectively reduced bad breath and inhibited bacteria causing halitosis. Thus, oral care products using *Sambucus williamsii* var. *coreana* extract should be developed and used for the improvement of oral health, as a viable alternative to mouthwashes with chemical formulations.
